# Case report: Substantial improvement of autism spectrum disorder in a child with learning disabilities in conjunction with treatment for poly-microbial vector borne infections

**DOI:** 10.3389/fpsyt.2023.1205545

**Published:** 2023-08-18

**Authors:** Amy Offutt, Edward B. Breitschwerdt

**Affiliations:** ^1^Heart and Soul Integrative Health, Marble Falls, TX, United States; ^2^Intracellular Pathogens Research Laboratory, Department of Clinical Sciences, and the Comparative Medicine Institute, College of Veterinary Medicine, North Carolina State University, Raleigh, NC, United States

**Keywords:** tics, infections, *Bartonella*, Lyme, PANS, OCD, autism, ADHD

## Abstract

Poly-microbial vector-borne infections may have contributed to neuropsychiatric symptoms in a boy diagnosed with autism spectrum disorder. Targeted antimicrobial treatment resulted in substantial improvement in cognitive (such as learning disabilities, focus, concentration) and neurobehavioral (such as oppositional, defiant, anti-social, disordered mood, immaturity, tics) symptoms.

## Introduction

Autism, or autism spectrum disorder (ASD), is a common and increasingly diagnosed entity among children in the United States (1). This complex neurodevelopmental disorder manifests with atypical social communication skills, and interactions consisting of restrictive, repetitive patterns of behavior. CDC’s MMWR data reports an ASD prevalence of 1 in 36 children in 2020 in the United States (2). Ratajczak proposed that this disorder is a consequence of genetic defects, with or without inflammation of the brain (3). Based upon the diverse and wide range of clinical histories in these children, he also postulated that inflammation could be the result of placental defects, blood–brain barrier immaturity, an abnormal maternal immune response *in utero*, prematurity, encephalitis, or toxic environmental exposures (3).

Considering the medical complexity associated with the wide array of clinical presentations, there is a substantial need for additional research into the causes of ASD neuropsychiatric conditions. A case published in 2019 reported significant improvement in a 14-year-old boy diagnosed with Pediatric Acute Onset Neuropsychiatric Symptoms (PANS), who was treated for infection with *Bartonella henselae* (4). Antimicrobial treatments directed at this bacterial pathogen resulted in a gradual return to baseline activities, resolution of his Bartonella-associated cutaneous lesions, and discontinuation of all psychiatric medications. We now describe a case involving a boy with developmental delays from an early age that was presumed to be associated with a congenital brain anomaly. Shifts in the constellation of his symptoms resulted in a high level of clinical suspicion for potential underlying infectious etiologies (5). In conjunction with the administration of antimicrobial therapy, the boy experienced a positive clinical response, including being moved from a long-term special education classroom setting to an age appropriate and grade level general classroom setting, based on standardized testing and school performance, in conjunction with dramatic improvement in cognitive functions, and ultimately acceptance to a 4-year college. In addition to these cognitive improvements, several other non-cognitive symptoms also significantly improved. This case report provides further support to the growing body of literature indicating that infectious triggers may contribute to neuropsychiatric disorders, including those in children who phenotypically present as academically challenged, or meet the criteria for autism spectrum disorder.

### Background

A 14 year-old boy was examined at the request of his mother, who accompanied him to the appointment. She provided a detailed medical, developmental, and educational history. During this initial visit, his mother reported that their entire family had been challenged for many years by her son’s neuropsychiatric issues that included a diagnosis of autism spectrum disorder. This patient’s mother had taken him to several other physicians seeking an explanation for the fluctuating nature of his neuropsychiatric symptoms. Before his birth, *in utero* ultrasound showed cerebellar hypoplasia, for which prognostically his future neurological function was unknown. At 6-months of age, the boy developed a neck-roll-like tic; at 8 months he began to crawl; and at 10 months fell down the stairs but did not sustain any injuries. He had some developmental delays, including first walking at 18 months of age. He struggled to fall asleep at night, and developed unusual behaviors, including atypical physical movements (flapping, falling out of chairs, requiring assistance with coordinated movements involving heights like navigating open stairs). When he was noted to have impulsivity and learning difficulties, attention deficit hyperactivity disorder (ADHD) was diagnosed at 5 years of age, after which he was given educational accommodations in school. Although his mother reported that she believed that he could have received a diagnosis of autism by the usual criteria, the description used to acquire educational accommodations was described by those evaluating him in his elementary school years as “other impairments.” However, the mother did not advocate strongly for a diagnosis of autism at that time because her son was already getting accommodations. As he grew older, swimming, riding a bike, and playing catch were very difficult activities, but were marginally achieved. He was diagnosed with pediatric acute onset neuropsychiatric syndrome at age 10. In June 2019, at 14 years of age, he was diagnosed as autistic and a pediatric neurologist (Children’s Medical Center of Dallas, Dallas TX) also diagnosed autoimmune encephalitis. On Module 4 of the ADOS-2 administered by his school in 2019, the patient’s communication score was 9 and his reciprocal social interaction score was 8, for a combined score of 17, which meets the ADOS-2 classification of Autism. The Autism Diagnostic Observation Schedule, Second Edition (ADOS-2), Module 4 is considered a “gold-standard” instrument for diagnosing autism spectrum disorder (ASD). Although ADOS-2 screening has a high degree of sensitivity, it also has low specificity due to a high rate of false positives among adults with psychosis. (6). Following approval of insurance coverage, he was treated with intravenous immunoglobulin in November 2019, after which his mother reported improvement in most of his symptoms. However, in January 2020, behavioral challenges increased, including the boy running away from home.

When this patient was presented to our practice (AO), his mother reported multiple symptoms, both past and present. The mother had maintained detailed notes about his day-to-day mental, physical and emotional status, including how he responded to various medical and behavioral interventions. She reported that the boy would have a flare of various behavioral issues during times when antibiotics were administered for streptococcal pharyngitis and borreliosis. During his 14-year history, multiple therapeutics and lifestyle interventions had been attempted to manage his symptoms, including, among others, amantadine, doxycycline, minocycline, amoxicillin, amoxicillin/clavulanate, clindamycin, IV ozone, gluten-free diet and a dairy-free diet. Following the introduction of a drug, most therapeutics, particularly antibiotics, were quickly discontinued due to intolerable side effects and flaring of behavioral symptoms.

Reading comprehension testing was low, placing him in special education with accommodations. His mother reported that he had recently failed the STAAR test, which is state-wide standardized testing administered by his school district. His social skills had continued to regress and he had developed an affinity for constantly playing video games. Defiant behavior had increased. His mother noticed that he had an increased craving for sugar and that eating foods that contain refined sugar seemed to make his symptoms even more intense. Obsessive and compulsive symptoms, which were part of his pediatric acute-onset neuropsychiatric syndrome, were quite prominent. His habit of eating a wide variety of foods when younger had transitioned to a state of food aversions and “picky” eating. Based upon vision assessment, prism lenses had been recommended, but he refused to wear glasses.

The mother also reported that his behavior tended to improve when he was given ibuprofen, administered most afternoons. He was currently taking hydroxyzine to help with sleep initiation, but he stated that he did not have trouble sleeping. He indicated that he was annoyed with his mother and felt depressed when she would not let him eat dairy foods. He had multiple episodes prior to and after the presentation during which he had behavioral outbursts and ran away from home multiple times. Child protective services were even brought in during one of these episodes because he made false claims after a running away incident. His mother was also quite concerned about his nutrition and low weight, just over 100 pounds. Historically, the patient did not have a known tick bite or any other specific environmental exposures, even though he lived in a midwestern state where vector-borne diseases are common.

From birth, his mother had chronic inflammatory symptoms that prompted her to suspect the possibility of a congenital infection. His mother’s symptoms included childhood onset anxiety, bedwetting, emotional lability, dysautonomia, migraine headaches, joint pains, numbness in her digits, Graves’ disease at 32 years old, and a 6-month eating disorder as a teenager. She had multiple tick bites around ages 7 to 8, but she believed that she had been exposed even earlier based on her family’s recollection of her childhood behaviors. She noted that she was having problems with mood and anxiety as early as age 3.

**Figure 1:**
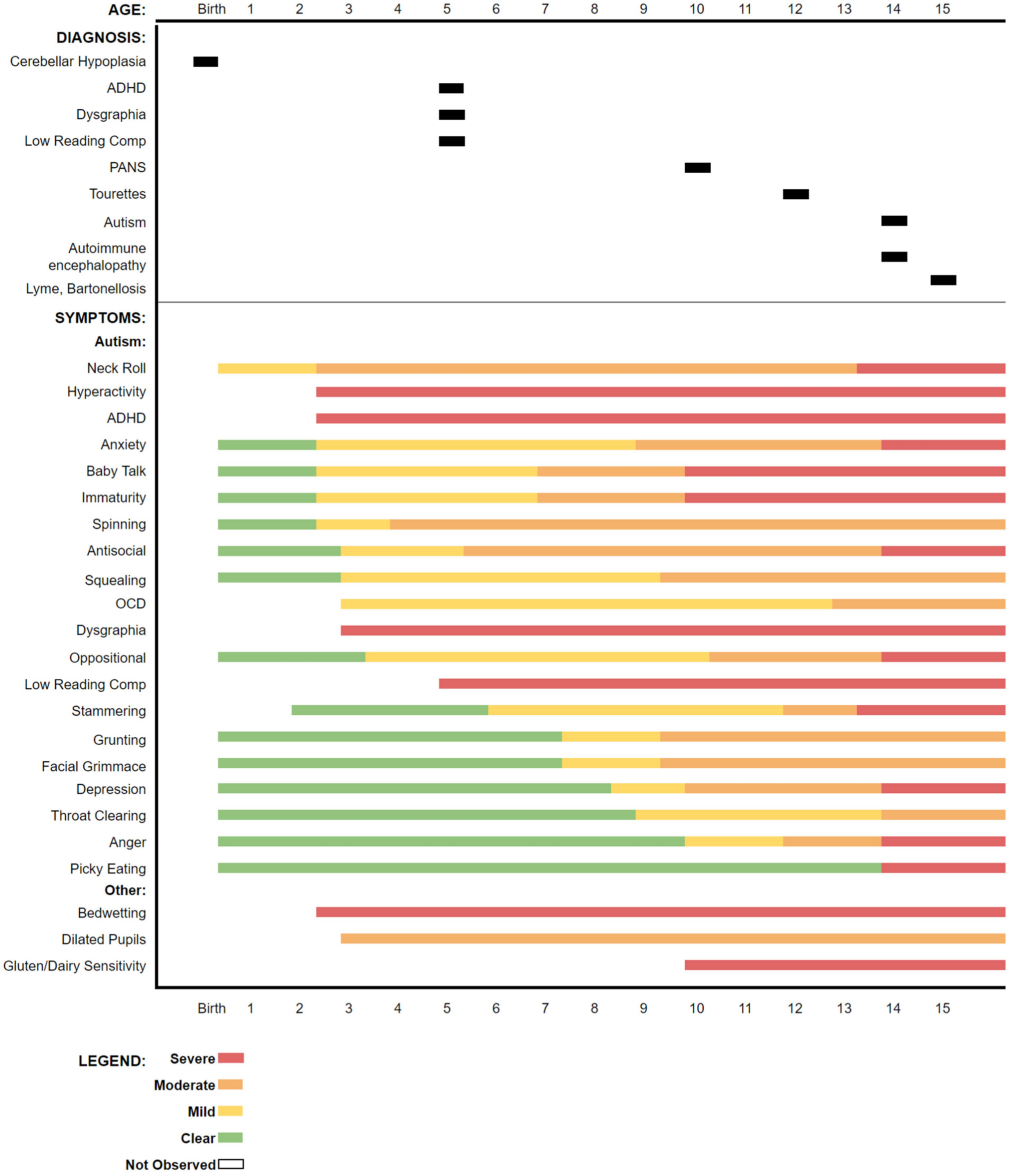
Timeline for diagnoses and symptom onset over the child’s lifetime as reported by the mother up to the time of initiating treatment for borreliosis at 15 years-old, approximately 9 months after presentation. Some symptoms were not recognized by the parents until the boy attended school.

The boy’s historical symptoms, up to the time of presentation, are summarized in a graphic timeline (Figure 1).

### Physical examination findings at initial presentation

Vital Signs: Blood Pressure (R arm): 100/62.

Weight: 101.6lbs.

Pertinent Findings: Generally thin habitus. The neuropsychiatric exam revealed slow, somewhat irregular speech cadence, frequently interruptive during conversation, diffuse, mildly reduced neuromuscular tone noted with gait generally intact, slightly depressed mood (negative outlook). Cardiovascular, pulmonary, and dermatologic exams were unremarkable.

### Patient laboratory data

Upon presentation, detailed medical records, including laboratory reports, were reviewed. Autoimmune, genetic, immunological, infectious disease, nutritional, and research testing results (7) are summarized in Table 1 in association with the time of testing, including the interpretation provided by the testing laboratory.

**Table 1:** Laboratory report results, reference ranges, and laboratory interpretation extracted from patient records and research testing.

**Age 10:**
IGeneX Laboratory:
*Borrelia burgdorferi*, IFA titer= 1:40 (reference range <40 negative, 40 equivocal, >=80 positive)*Borrelia burgdorferi* IgG and IgM = negative
*Bartonella henselae* IgG and IgM = negative
*Babesia microti* IgM = negative, IgG = 1: 40 (may or may not suggest active infection) *Babesia duncani* IgG / IgM = negative
*Babesia* FISH = negative
Cunningham Panel, (Moleculera Laboratory):
Anti-tubulin = 1000 (upper limit of normal range, 0 - 1000)
Four other measured parameters were within normal limits.
LabCorp:
Anti-DNase B Strep Antibodies = 1020 U/ml (reference range 0 - 170)Antistreptolysin O Antibodies - 620.8 IU/ml (reference range 0 - 200)Beta Strep Gp A Culture = negative
Nutritional deficiencies = thiamin, riboflavin, magnesiumComprehensive Stool Profile = Dientamoeba fragilis, dysbiosis
Multiple IgE elevations - cat dander, dog dander, shrimp, cockroachElevated IgG subclass 4 (164, range 1 - 121)
MTHFR C677T single mutation
**Age 14:**
Mayo Clinic Laboratories:Autoimmune Encephalopathy, GAD65 Ab, Assay, S: 0.14 nmol/L (reference range <=0.02)
**Age 16 (after treatment with disulfiram, minocycline, and rifampin):**
Vibrant America Lab *Borrelia burgdorferi* and *Bartonella henselae* testing
VlsE1 IgM elevated (IgG normal)
C6 peptide = IgM elevated (IgG normal)
p18 (DpbB) = IgM elevated
P45 = IgG elevated
Crude extract B31 = IgM elevated
Crude extract 297 = IgM elevated
Overall laboratory interpretation for Lyme disease serological testing: NEGATIVE
• *Borrelia afzelli* BmpA = IgG elevated
• *Borrelia garinii* DbpA = IgG elevated
• *Borrelia spielmanii* DbpA = IgM elevated
• *Bartonella henselae* SucB = IgG elevated
**Age 17:**
In August 2022, the patient and his mother entered a research study entitled Detection of *Bartonella* Species in the Blood of Healthy and Sick People (North Carolina State University [NCSU] Institutional Review Board approval, IRB 1960). Both study participants provided three blood and serum collections, obtained on alternate days, and gave permission for testing for *Babesia, Bartonella* and *Borrelia* species. Previously described serology and enrichment blood culture qPCR and droplet digital PCR (ddPCR) assays were used for testing (7).
Research Testing, (North Carolina State University Intracellular Pathogens Research Laboratory).
*Bartonella* IFA serology:
Patient
• *Bartonella vinsonii* subsp. *berkhoffii* Genotype I < 1:16
• *Bartonella vinsonii* subsp. *berkhoffii* Genotype II < 1:16
• *Bartonella henselae* < 1:16
• *Bartonella koelerae* < 1:16
• *Bartonella quintana* < 1:16
Patient’s mother
• *Bartonella vinsonii* subsp. *berkhoffii* Genotype I < 1:16
• *Bartonella vinsonii* subsp. *berkhoffii* Genotype II < 1:16
• *Bartonella henselae* < 1:16
• *Bartonella koelerae* < 1:16
• *Bartonella quintana* < 1:16
Interpretation: Patient and patient’s mother seronegative to all five IFA antigens
qPCR and ddPCR testing was performed on blood, serum, 7, 14, and 21-day enrichment blood culture samples (24 independent DNA extractions per individual tested) for the patient and patient’s mother.
qPCR and Droplet Digital PCR (ddPCR) Testing Results:
Patient
• qPCR
◦ *Babesia* = Negative
◦ *Bartonella* = Negative
◦ *Borrelia* = Negative
• ddPCR
◦ *Babesia* = Negative
◦ *Bartonella* = *Bartonella quintana* (DNA amplified and sequenced from a 21-day enrichment blood culture
◦ *Borrelia* = one ddPCR+ droplet in 4/24 samples tested, unable to confirm by DNA sequencing
Patient’s mother
• qPCR
◦ *Babesia* = Negative
◦ *Bartonella* = Negative
◦ *Borrelia* = Negative
• ddPCR
◦ *Babesia* = Negative
◦ *Bartonella* = *Bartonella koehlerae* (DNA amplified and sequenced from a 21-day enrichment blood culture
◦ *Borrelia* = one ddPCR+ droplet in 3/24 samples tested, unable to confirm by DNA sequencing

In summary, prior to presentation to our practice, this 14-year-old boy had been diagnosed with cerebellar hypoplasia (*in utero*), ADHD (onset age 5 years), PANS (onset during elementary school, but not officially diagnosed until 10 years-old), autism (symptoms onset during elementary school, but not officially diagnosed until 14 years-old), and autoimmune encephalopathy (diagnosed at age 14 years). After our medical evaluation, partially treated Lyme disease (borreliosis), with chronic neurocognitive effects, and congenital systemic bartonellosis were tentatively diagnosed.

### Assessment and treatment plan

**Figure 2:**
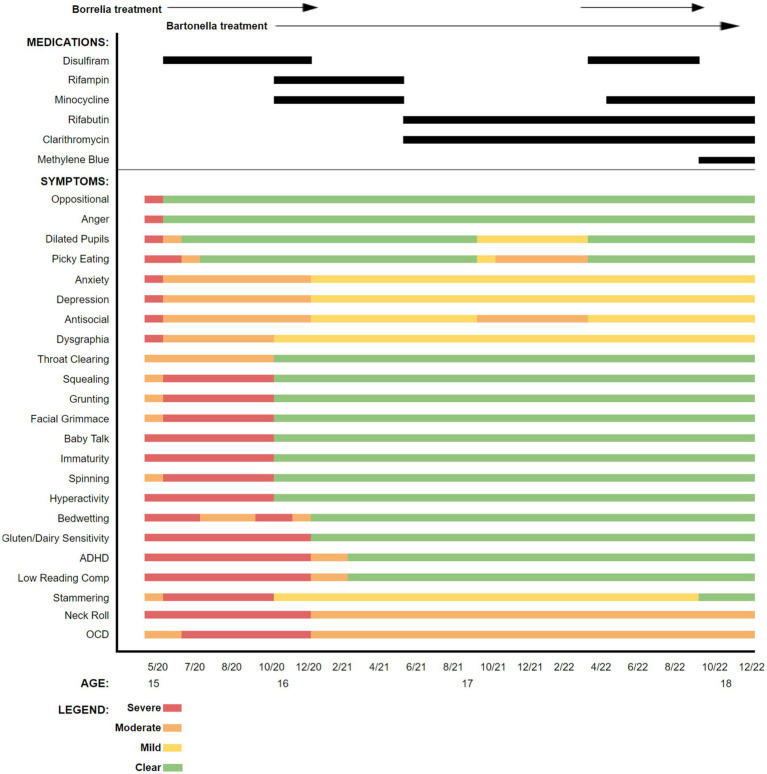
Symptoms reported at the time of presentation to our practice and following initiation of each antibiotic or antibiotic combination. Symptoms and clinical signs were assessed by the mother as mild, moderate, severe, or cleared.

Based upon the boy’s historical symptoms, prior laboratory testing results, and previous intolerance to multiple therapeutics, treatment initially targeted borreliosis and then bartonellosis. Disulfiram was initiated because the drug had not been tried previously, had somewhat predictable and manageable side effects, and prior therapeutic agents had not been well tolerated. Also, recent *in vitro* data and patient studies supported the potential efficacy of disulfiram for treating borreliosis (8–17). A graphic representation of symptom tracking in conjunction with the administration of disulfiram and the other subsequently administered antibiotics is provided in Figure 2. As was done prior to antibiotic administration, symptoms, carefully monitored and documented in writing by his mother, were used to generate figures for both pre-and post-antibiotic treatment periods.

During treatment, certain interventions including changes in antibiotics, resulted in abrupt or, sometimes, more gradual changes in symptoms. As the patient’s symptoms would plateau, a transition to a new therapeutic regimen was implemented, gradually administering individual or combinations of multiple different antimicrobial agents, including disulfiram, rifampin, minocycline, rifabutin, clarithromycin, and methylene blue (18–20). Supportive measures including probiotics, antioxidants, and anti-inflammatories were administered throughout the course of care. Again, patient responses during the time course of treatment are summarized in the bar graph (Figure 2).

#### Treatment outcome

This patient had a cadre of symptoms documented by his mother with all being present to some extent just prior to or at the time of initiation of antibiotic treatment. Oppositional behavior and anger improved almost immediately after the initiation of disulfiram and these symptoms remained clear throughout the treatment period. Other symptoms initially persisted but lessened in severity. Still other symptoms, including squealing, grunting, grimacing, and stammering worsened when disulfiram was introduced. However, because of noticeable improvement in some of the other symptoms, antibiotics were continued despite the worsening of other symptoms. As is demonstrated in the graphic representation, several antimicrobials were selected, and some were only utilized once, while others were initiated, discontinued, and reinstated, based on clinical responses. Shortly after treatment was initiated, the patient’s appetite improved resulting in a 35 pound weight gain over the first 6 months, potentially addressing prior concerns about his overall nutritional status.

As of December 2022, many of the boy’s chronic symptoms had resolved or improved. One of the most noticeable improvements by his parents, his teachers, and school administrative staff was that his academic testing transitioned from the special education level to tenth grade level, as he no longer required the special accommodations that had been provided at his school. His IQ testing increased by 7 points and his English STAAR testing scores increased by 27 percentage points, placing him in the 46th percentile for 10th grade (Table 2). Also, after initiation of antimicrobial drug administration in June 2020, he experienced substantial improvement in structured educational testing scores for reading and mathematics (Table 2). With these significant improvements, his special education placement ended, and he transitioned to a regular classroom setting where he was able to maintain adequate academic performance through high school graduation. In addition, there was a sudden and noted improvement in his social skills, progress that had not been noted by school officials since kindergarten. He has been able to take a college entrance examination resulting in acceptance to a 4-year university. This accomplishment was not something that his family or educators would have anticipated before May of 2020.

**Table 2:** Results of academic assessments included structured IQ testing administered by the school neuropsychologist, as well as standardized educational assessments. After antimicrobials were administered to treat Bartonellosis and Borreliosis, based upon WISC testing, the patient’s IQ rose 7 points between 2012 and 2021. NOTE: The higher KABC II NU score in 2016 should not be directly compared to the WISC and WAIS testing scores due to the difference in testing approach. The KABC II NU testing methodology differs by measuring intelligence without the Short-term Memory and Processing Speed components that are often depressed in students with autism, ADHD, or emotional disturbances. Results of standardized educational assessments for reading and mathematics for grades 3 through 11 show an increase in English performance by 27 percentage points and an increase in math performance by 9 percentage points after treatment. Antimicrobial drug administration was initiated in June 2020.

IQ SCORES				
WISC IV* 2012 (2nd grade)	KABC II NU** 2016 (6th grade)	WISC V/ CTOPP for Auditory Processing*** 2018 (8th grade)	WAIS IV**** 2021 (11th grade)	
88	97	89	95	
STRUCTURED EDUCATIONAL ASSESSMENTS				
	**ENGLISH**		**MATH**	
**Grade (Year)**	**Percentile**	**Comments**	**Percentile**	**Comments**
**Missouri Assessment Program (MAP)**				
3rd (2014)		Basic		Basic
4th (2015)		Below Basic		Basic
5th (2016)		Below Basic		Below Basic
6th (2017)		Basic		Basic
7th (2018)		Basic		Proficient
**Texas STAAR Assessment**				
8th (2019)	19	Did not meet grade level	49	Approaches grade level
10th (2021)	46	Approaches grade level		*Not administered*
**Measures of Academic Performance (MAP)**				
11th (2022)		*Not administered*	58	Grade level

* WISC: Wechsler Intelligence Scale for Children.

** KABC II NU: Kaufman Assessment Battery for Children 2 Normative Update.

*** WISC V/ CTOPP for Auditory Processing: Wechsler Intelligence Scale for Children V/Comprehensive Test of Phonological Processing.

**** WAIS: Wechsler Adult Intelligence Scale.

Below Basic: Does not show understanding of the content expected at this grade level.

Basic: Demonstrates a partial understanding of the content expected at this grade level.

Proficient: Demonstrates understanding of the content expected at this grade level.

As of December 2022, the patient has maintained improvements as noted in the symptom timeline, but he is unable to discontinue antimicrobials without a noticeable recurrence of several of his symptoms. This affects his quality of life significantly, and therefore, we have opted to continue antimicrobial coverage while considering additional strategies.

## Discussion

Autism spectrum disorders are increasing in prevalence and are negatively impactful on many families and individual communities throughout the United States. The CDC estimates that 1 in 36 children in the United States were diagnosed with an autism spectrum disorder in 2020 (2). The estimated annual cost for children’s autism spectrum disorder care is between $61 and $66 billion, while the annual adult services cost is estimated to be between $175 and $196 billion (21). A strategic therapeutic approach that significantly addresses the burdens placed on these families, their communities and society in general has been elusive. Despite substantial research efforts and an ever-increasing need to better understand the cause(s) of ASD, effective therapeutic interventions have focused on modifying or controlling abnormal behaviors. With the current medical philosophy that these disorders are largely caused by genetic abnormalities, the extent to which infectious agents might be contributory has been minimally explored on a clinical or research basis. It stands to reason that exposure to infectious agents or neurotoxins, either *in utero* or later during the early developmental years, might result in mutations or alternatively impact disease expression in genetically predisposed individuals. Agents that induce persistent, stealth intravascular or central nervous system infections would potentially negate the effectiveness of symptomatic therapies.

This patient had a well-defined embryonic abnormality diagnosed *in utero*, clearly complicating the developmental assessment of his physical and mental capabilities. Based upon symptomatology he was diagnosed and treated medically for over a decade as an “impaired” child, up to the time of our initial evaluation. Test results in this patient supported exposure to one or more vector borne pathogens, a serological diagnosis of bartonellosis and borreliosis, and the borderline presence of anti-neuronal antibodies, which can occur in association with vector borne and other non-vector borne infections (22). Therefore, a decision was made to treat with antimicrobial agents targeting a potential infectious etiology, with secondary auto-antibody formation. His response to this therapeutic approach included improvement or elimination of a substantial number of neuropsychiatric symptoms and significant improvement in his academic status, placing him at grade level without accommodations and eligible for more advanced educational planning. Lacking the benefit of clinical trials or previously defined treatment protocols, antibiotics targeting two vector borne genera were administered for an extended period, while carefully monitoring various symptomatic responses and safety parameters. Despite having stable or progressive symptoms for potentially his entire life, the response to treatment suggested that neither the cerebellar hypoplasia, inherited genetic defects, or autoimmunity were singularly causative. Strikingly, normalization of some symptoms supported the possibility that there had been no permanent CNS damage, although the onset and duration of the putative infections remains unknown. Although very much incompletely studied, perinatal transmission of both *Bartonella* and *Borrelia* species have been suspected (23, 24). Ongoing antimicrobial coverage was required at the time of writing this case report due to recurrence of all symptoms to one degree or another with the exception of oppositional behavior and anger with discontinuation, potentially related to ongoing infection with Borrelia and *Bartonella quintana*. Treatments with single or combinations of several antimicrobial drugs were accompanied by substantial improvements in neurological symptoms; however, research testing in August, 2022, documented the presence of *B. quintana* and potentially Borrelia spp. DNA in blood and enrichment blood cultures by ddPCR and qPCR/DNA sequencing testing in the patient and his mother. Our interpretation is that the treatments utilized suppressed, but did not eliminate these infections in the patient, indicating the need for future studies that use sequential testing with advanced direct detection modalities to assess treatment response. Nonetheless, until further documented efficacious therapeutic options are available, and with close monitoring for safety and tolerability, benefits from improved quality of life outweigh the risks of long-term antibiotic administration.

We propose that a risk/benefit analysis in most patients diagnosed with autism spectrum disorder would warrant evaluation and treatment for potential chronic infectious triggers. Incorporation of diagnostic modalities that detect pathogen antigens or DNA will allow for increased support for a causative role for specific organisms in association with patient symptoms. More research is obviously needed, but if this type of clinical and microbiological approach were to be further explored, the result could dramatically impact future clinical practice. Documenting the presence of an infectious etiology in blood or cerebrospinal fluid could provide an even more meaningful, positive impact in the lives of so many who are suffering with limited therapeutic options. The fact that this patient was treated as a teen also indicates that these infections may persist, well beyond any acute exposures, or occurring *in utero*, or during the perinatal period. Therefore, treating earlier in life may result in improvements in academic performance, improved social development, and a better chance at full adult independence, which would represent a significantly altered outcome for many people. Currently, borreliosis and bartonellosis are infections that are rarely considered in the evaluation and treatment of patients with neuropsychiatric presentations, but this case and others point to the possibility that chronic persistent infections may contribute to the presence and severity of symptoms in children and young adults with neuropsychiatric symptoms. Although an autoimmune or immunomodulatory component is suspected, more research is clearly needed to further delineate if it is the infection itself or the secondary immune consequences of the infection that may contribute to this phenotypic presentation of neuropsychiatric disease.

## Conclusion

This teenage boy had a drastic improvement in his neuropsychiatric symptoms and in his academic standing, moving from special education services with accommodations to grade level academic standing without accommodations, to college acceptance. Progressive symptomatic improvement occurred only following targeted administration of antimicrobial agents directed at suspected, underlying, chronic infectious pathogens, namely the causative agents of bartonellosis and borreliosis. Further research is clearly needed to define if or the extent to which occult infections can contribute to neuropsychiatric illness, such as ASD.

## Data availability statement

The original contributions presented in the study are included in the article/supplementary material, further inquiries can be directed to the corresponding author.

## Ethics statement

The patient and his parents provided their written informed consent to participate in this study. Written informed consent was obtained from the individual(s) for the publication of any potentially identifiable images or data included in this article. Research testing at North Carolina State University was conducted as a component of a study entitled: Detection of *Bartonella* Species in the Blood of Healthy and Sick People (NCSU Institutional Review Board approval, IRB 1960).

## Author contributions

AO was the primary physician providing medical care, was in charge of the diagnostic testing, therapy plan, patient follow-up, and drafted the manuscript. EB conducted further microbial diagnostic testing on the patient and co-wrote the manuscript. All authors contributed to the article and approved the submitted version.

## Funding

Research testing was supported through a grant from the Steven and Alexandra Cohen Foundation and donations to the Bartonella/Vector Borne Diseases Research Fund at the North Carolina State University College of Veterinary Medicine and by the state of North Carolina.

## Conflict of interest

AO was employed by Heart and Soul Integrative Health.

The remaining author declares that the research was conducted in the absence of any commercial or financial relationships that could be construed as a potential conflict of interest.

## Publisher’s note

All claims expressed in this article are solely those of the authors and do not necessarily represent those of their affiliated organizations, or those of the publisher, the editors and the reviewers. Any product that may be evaluated in this article, or claim that may be made by its manufacturer, is not guaranteed or endorsed by the publisher.
